# Supplementary material for: Detection of hidden antibiotic resistance through real-time genomics
Source: Nat Commun. 2024 Jun 28;15:5494. doi: 10.1038/s41467-024-49851-4 (PMC11214615; doi:10.1038/s41467-024-49851-4)
Supplement: Supplementary file 1 — Supplementary Information [file 41467_2024_49851_MOESM1_ESM.pdf]

Detection of hidden antibiotic resistance through real-time genomics

Supplementary Information

Ela Sauerborn[1,2,3,4], Nancy Carolina Corredor[4], Tim Reska[1,2,3], Albert Perlas[1,2], Samir Vargas da Fonseca, Atum[1,25,6], Nick Goldman[7], Nina Wantia[4], Clarissa Prazeres da Costa[4,8,9], Ebenezer Foster-Nyarko[10], Lara Urban[1,2,3*]

[1]Helmholtz AI, Helmholtz Zentrum Muenchen, Neuherberg, Germany
[2]Helmholtz Pioneer Campus, Helmholtz Zentrum Muenchen, Neuherberg, Germany
[3]Technical University of Munich, School of Life Sciences, Freising, Germany
[4]Institute of Medical Microbiology, Immunology and Hygiene, TUM School of Medicine and Health, Technical University of Munich, Germany
[5]Departamento de Química Fundamental, Instituto de Química, Universidade de São Paulo, São Paulo, Brazil
[6]Departamento de Bioquímica, Instituto de Química, Universidade de São Paulo, São Paulo, Brazil
[7]European Molecular Biology Laboratory, European Bioinformatics Institute (EMBL-EBI), Wellcome Genome Campus, Cambridge, UK
[8]Center for Global Health, TUM School of Medicine and Health, Technical University of Munich, Munich, Germany
[9]German Center for Infection Research (DZIF), partner site Munich, Germany
[10]Department of Infection Biology, London School of Hygiene & Tropical Medicine, Keppel Street, London, UK

*Corresponding author: lara.h.urban@gmail.com

This file includes:

- Supplementary Table 1
- Supplementary Table 2
- Supplementary Figure 1

Supplementray Table 1. Real-time genomic sequencing and assembly metrics before and after treatment with CAZ-AVI. Genomic material of each bacterial isolate was sequenced for 15 hours; the pre-treatment isolate was additionally sequenced for a further 8 hours to simulate adaptive sequencing in the clinical setting.

Genomic sequencing and assembly metrics	Pre-treatment sample		Post-treatment sample
	15h	+8h	15h
Median read quality (Phred)	12.3	13.0	12.3
Median read length [kb]	4.1	5.3	4.5
Total number of bases [Mb]	442	1,551	194
Assembly coverage (median)	68	271	30
Number of contigs (>50 kb)	5	5	5
Contig N50 [Mb]	5.2	5.2	5.3

Supplementary Table 2. MIC measurements with VITEK2 and Liofilchem®MIC test strip for CAZ-AVI. The colouring scheme indicates the interpretation of MICs according to EUCAST guidelines (green: susceptible/intermediate; red: resistant).

Antibiotic	Pre-treatment isolate MIC (mg/L)	Post-treatment isolate MIC (mg/L)
Ampicillin	>32	>32
Ampicillin-Sulbactam	>32	>32
Piperacillin	>128	>128
Piperacillin-Tazobactam	>128	>128
Cefuroxime	>64	>64
Cefotaxime	>64	>64
Ceftriaxone	>64	>64
Ceftazidime	>64	>64
Imipenem	>16	1
Meropenem	>16	2
Ertapenem	>8	>8
Gentamicin	>16	>16
Ciprofloxacin	>4	>4
Ceftazidime-Avibactam	2	>256

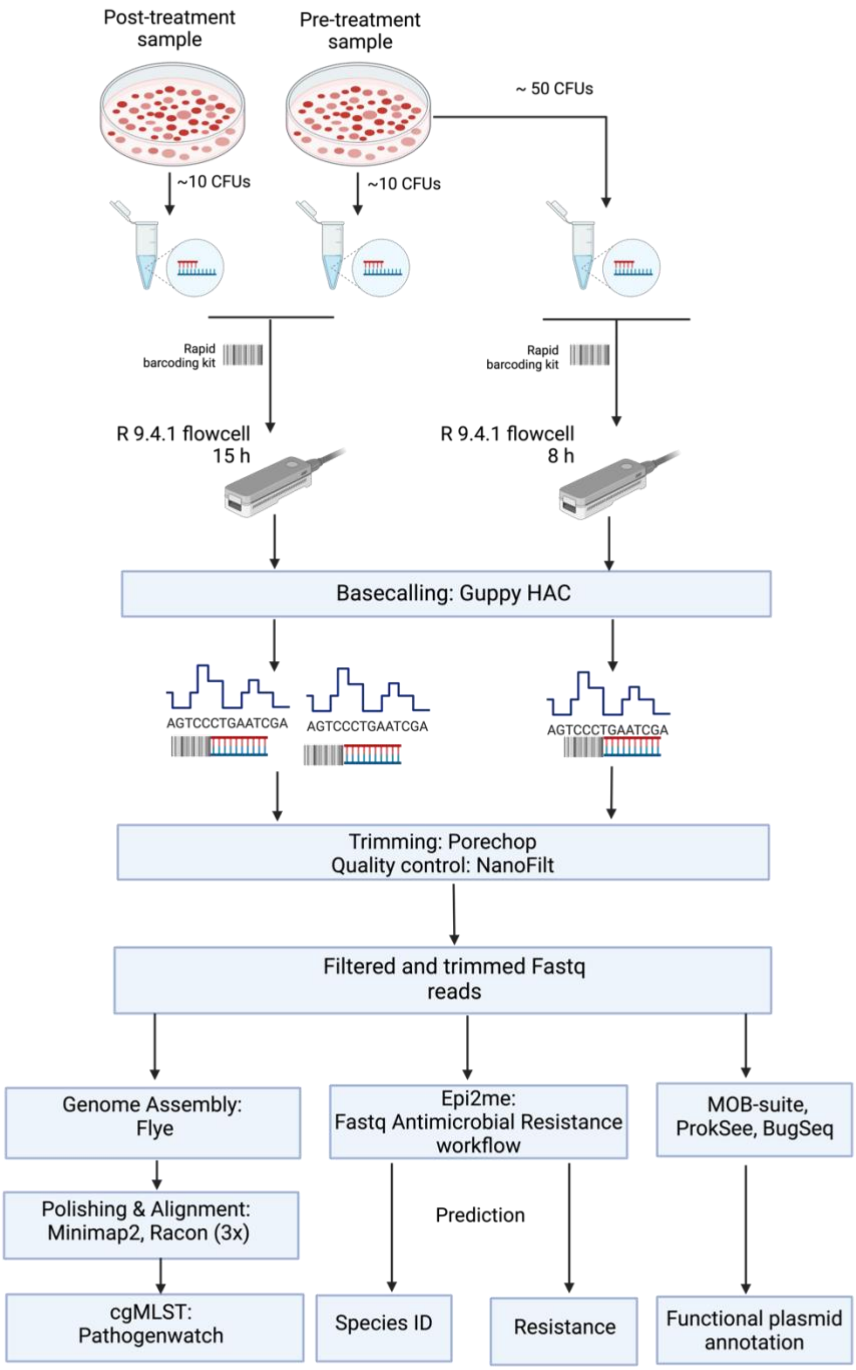

Supplementary Figure 1. Bioinformatic workflow for species identification and antibiotic resistance prediction from nanopore sequencing data of pre- and post-treatment isolates (HAC: High-accuracy basecalling; ID: Identification; cgMLST: core genome Multi-Locus Sequence Typing). All computational analyses were conducted on a portable laptop with an 8 GB NVIDIA GeForce RTX 4070 GPU, 16 GB 5200 MHz RAM, and an Intel i7-13800H CPU with 14 cores and 20 threads.
