# Supplementary material for: Detection of hidden antibiotic resistance through real-time genomics
Source: Nat Commun. 2024 Jun 28;15:5494. doi: 10.1038/s41467-024-49851-4 (PMC11214615; doi:10.1038/s41467-024-49851-4)
Supplement: Supplementary file 3 — Description of Additional Supplementary Files [file 41467_2024_49851_MOESM3_ESM.pdf]

Description of Additional Supplementary Files

Supplementary Data 1 : Antimicrobial resistance genes detected by EPI2ME's Fastq Antimicrobial Resistance workflow based on the Antimicrobial Resistance protein homolog model with a minimum detection accuracy threshold of 90% (Methods). *Pre-treatment isolate* sheet: Antimicrobial resistance genes detected in the first and second sequencing round of the pre-treatment isolate; *Post-treatment isolate* sheet: Antimicrobial resistance genes detected in the sequencing round of the post-treatment isolate.
